# Exploring the neural mechanisms of mild cognitive impairment in elderly patients with coronary artery disease using machine learning and source-localized EEG

**DOI:** 10.3389/fneur.2026.1766328

**Published:** 2026-04-10

**Authors:** Huiwei Wan, Yanyao Deng, Fangbo Lin, Jie Li, Hao Xiao, Le Xiao

**Affiliations:** Department of Rehabilitation, The First Hospital of Changsha, Changsha, Hunan, China

**Keywords:** coronary artery disease, electroencephalography, functional connectivity, lagged phase synchronization, machine learning, mild cognitive impairment, source reconstruction

## Abstract

**Objective:**

This study seeks to investigate the electrophysiological mechanisms associated with mild cognitive impairment (MCI) in elderly patients with coronary artery disease (CAD) through the application of source-reconstructed EEG in conjunction with machine learning methodologies.

**Methods:**

We retrospectively analyzed clinical data and resting-state 64-channel EEG recorded during hospitalization at The First Hospital of Changsha. Participants included primary hypertension without CAD (*n* = 53) and CAD with primary hypertension (*n* = 117), with CAD stratified by Montreal Cognitive Assessment (MoCA) into MCI (*n* = 49) and cognitively normal (*n* = 68). EEG sources were reconstructed using an ICBM152-based head model and BEM forward modeling, yielding 82 Brodmann-atlas ROIs; functional connectivity was quantified using lagged phase synchronization (LPS) in delta (0.5–4 Hz), theta (4–8 Hz), alpha (8–13 Hz), and beta (13–30 Hz) bands. Group comparisons applied false discovery rate correction. For MCI classification among patients with CAD, the dataset was randomly split into training and testing sets (7:3). Feature selection was performed in the training set using an independent-samples *t*-test followed by L1-penalized logistic regression. Subsequently, eight machine-learning classifiers were trained using the selected LPS features, with hyperparameters optimized by grid search under five-fold cross-validation. Model interpretability was assessed using SHAP.

**Results:**

Baseline demographics and vascular comorbidities were comparable across groups; MoCA scores were lower in the MCI subgroup. Relative to hypertensive controls without CAD, cognitively normal CAD patients showed reduced frontal connectivity, including decreased alpha-band LPS (BA8L–46R) and beta-band LPS (BA44L–44R). Compared with cognitively normal CAD, CAD with MCI exhibited broader multi-band dysconnectivity across alpha, beta, theta, and delta bands, with mixed delta-band changes. In the test set, the Gradient Boosting model achieved the best performance for identifying MCI within CAD (AUC = 0.895). SHAP highlighted the most influential features, led by decreased alpha-band BA8L–46R connectivity, alongside delta- and beta-band alterations.

**Conclusion:**

Coronary artery disease is associated with frontal network disruption, which becomes more extensive and frequency-diverse as MCI progresses. Interpretable machine learning further highlights a small set of connectivity abnormalities—particularly within premotor–prefrontal pathways—as candidate markers for MCI classification within a CAD cohort, supporting a vascular-relevant interpretation, which warrants further validation.

## 1 Introduction

Coronary artery disease (CAD) remains a leading cause of morbidity and mortality worldwide, affecting over 126 million individuals globally and contributing to approximately 9 million deaths annually (1). In elderly populations, CAD not only impairs cardiovascular function but also heightens the risk of cognitive decline, with mild cognitive impairment (MCI) emerging as a prevalent comorbidity that precedes more severe dementias and significantly diminishes quality of life (2).

Coronary artery disease involves progressive atherosclerotic narrowing of the coronary arteries, leading to myocardial ischemia and systemic vascular dysfunction. In older adults, this vascular burden is relevant to brain health because endothelial dysfunction and impaired autoregulation can plausibly constrain cerebral perfusion and amplify neuroinflammatory and oxidative pathways linked to cognitive vulnerability. MCI represents an early and clinically actionable stage, defined by measurable deficits in memory, attention, or executive function with relative preservation of daily independence. EEG is well-suited to this context because it captures millisecond-scale network dynamics that may be sensitive to vascular stressors. Accordingly, recent EEG work in MCI has broadened from band-power summaries to more explicit descriptions of network organization. Graph-theoretical analyses applied to functional connectivity matrices have reported topology consistent with reduced large-scale integration and altered segregation in MCI compared with cognitively healthy controls, often in slower frequencies (e.g., lower global efficiency with accompanying changes in clustering or path-based metrics) (3, 4). Microstate studies have also described reproducible shifts in the temporal expression of canonical scalp topographies in MCI, including differences in microstate occurrence/coverage and transition patterns (5). In parallel, individualized frequency markers such as dominant or peak alpha frequency frequently show slowing in MCI phenotypes, while frequency-variability findings are more heterogeneous across cohorts, suggesting sensitivity to subtype and measurement choices (6). More recently, separating periodic oscillations from the aperiodic 1/f background has helped clarify that apparent band differences can partly reflect broadband spectral tilt rather than true oscillatory changes (7). Taken together, these approaches support the concept that MCI is accompanied by quantifiable network-level disruption, but they also underscore that effect direction and reproducibility depend strongly on how connectivity is estimated and how spectral features are defined.

Additionally, a persistent limitation in connectivity-based EEG studies is contamination by volume conduction and common-source mixing, which can inflate zero-lag coupling and complicate anatomical inference, particularly when translating from sensor space to source space. This is one reason why lag-based connectivity measures are increasingly used when the goal is to relate coupling patterns to disease-relevant network disruption rather than to field spread. Lagged phase synchronization (LPS) directly targets non-zero-lag phase relationships and reduces the influence of instantaneous mixing, providing a more conservative estimate of functional coupling that is well-matched to source-localized analyses (8). In a CAD cohort, where systemic vascular dysfunction may plausibly perturb fronto-parietal control networks and their frequency-specific coordination, focusing on source-level LPS offers a pragmatic compromise: it retains EEG’s temporal sensitivity, supports anatomically grounded hypotheses, and helps align the connectivity metric with the intended biological interpretation.

Despite these insights, the specific neural correlates of MCI within CAD populations remain underexplored, particularly in the context of comorbid hypertension, a common confounder in elderly cohorts. Existing research predominantly focuses on structural imaging or functional MRI, which offer limited temporal resolution and overlook the millisecond-scale dynamics crucial for understanding oscillatory disruptions (9). EEG studies in CAD have reported global connectivity changes, but few integrate source localization to pinpoint regional impairments or employ interpretable ML to quantify feature contributions to cognitive decline (10). For instance, while some reports highlight frontal lobe hypoconnectivity in vascular dementia, they rarely differentiate MCI stages or control for hypertension’s independent effects on EEG (11). This gap hinders the development of targeted biomarkers, as it leaves unresolved the specific connectivity alterations within distinct frequency bands that are unique to CAD-MCI diagnostic models. Moreover, methodological limitations, such as small sample sizes and lack of multiple comparison corrections, compromise the reliability of prior findings, leaving a need for rigorous, integrated approaches to elucidate CAD’s electrophysiological impact on cognition.

This study aims to investigate the neural mechanisms of MCI in elderly CAD patients by analyzing source-localized EEG functional connectivity and constructing ML-based diagnostic models. Specifically, we hypothesize that CAD may be associated with reduced high-frequency LPS within frontal networks, and that MCI status within CAD is associated with more widespread multi-band disruptions, consistent with progressive network fragmentation in a vascular-risk context (12). To test this, we compare EEG features between CAD patients with and without MCI, as well as non-CAD hypertensive controls, to identify differential impairments. Additionally, we develop and evaluate ML classifiers to predict MCI status, using SHapley Additive exPlanations (SHAP) values to interpret the contributions of EEG features. These objectives seek to provide novel biomarkers for early MCI detection in CAD, emphasizing practical applications in clinical screening and interdisciplinary insights into vascular-neural interactions.

## 2 Methods

This study retrospectively analyzed clinical information and EEG data from patients with primary hypertension and from patients with primary hypertension comorbid with CAD who were recruited at The First Hospital of Changsha between January 2023 and December 2024 and met the inclusion and exclusion criteria. CAD patients were subsequently stratified into an MCI group and a non-MCI group according to the Montreal Cognitive Assessment (MoCA). The study was conducted in accordance with the Declaration of Helsinki and was approved by the Ethics Committee of The First Hospital of Changsha (2024–58).

First, differences between cognitively normal patients with primary hypertension plus CAD and patients with primary hypertension without CAD were compared to preliminarily examine CAD-associated impairment in brain electrophysiological function. Next, brain electrophysiological activity was further compared between CAD patients with MCI and cognitively normal CAD patients to investigate the neural mechanisms underlying MCI in CAD. Finally, based on electrophysiological differences between CAD patients with and without MCI, a diagnostic model was developed to interpret the contribution of distinct EEG abnormalities to MCI identification in CAD and to delineate the most specific brain functional impairments associated with CAD-related MCI.

### 2.1 Inclusion and exclusion criteria

#### 2.1.1 Inclusion criteria for primary hypertension group

(1) Diagnosis of primary hypertension in accordance with the “2018 ESC/ESH Guidelines for the management of arterial hypertension” (13); (2) Age ≥ 60 years and <80 years; (3) Cognitively normal as evaluated by MoCA (Score ≥ 26); (4) EEG data collected during hospitalization; (5) Complete preservation of data related to the study in medical records.

#### 2.1.2 Exclusion criteria for primary hypertension group

(1) History of diseases potentially causing cognitive impairment such as stroke; (2) Presence of other mental or neurological diseases affecting cerebral electrical activity; (3) Use of medications affecting cerebral electrical activity such as sedatives and hypnotics during hospitalization; (4) Receipt of interventions affecting cerebral electrical activity such as neuromodulation during hospitalization.

#### 2.1.3 Inclusion criteria for CAD group

(1) Diagnosis of chronic CAD in accordance with the “2023 AHA/ACC/ACCP/ASPC/NLA/PCNA Guideline for the Management of Patients With Chronic Coronary Disease: A Report of the American Heart Association/American College of Cardiology Joint Committee on Clinical Practice Guidelines” (14); (2) Diagnosis of primary hypertension in accordance with the “2018 ESC/ESH Guidelines for the management of arterial hypertension” (13); (3) Age ≥ 60 years and < 80 years; (4) Evaluated as MCI or cognitively normal using the Montreal Cognitive Assessment scale during hospitalization; (5) EEG data collected during hospitalization; (6) Complete preservation of data related to the study in medical records.

#### 2.1.4 Exclusion criteria for CAD group

(1) Participants with comorbid neurological or systemic conditions that could confound cognitive assessment, such as stroke, were excluded; (2) Presence of other mental or neurological diseases affecting cerebral electrical activity; (3) Use of medications affecting cerebral electrical activity such as sedatives and hypnotics during hospitalization; (4) Receipt of interventions affecting cerebral electrical activity such as neuromodulation during hospitalization; (5) History of cardiovascular surgery.

### 2.2 EEG acquisition parameters

The EEG signals of all included subjects were collected using a 64-channel Neuroscan SynAmps RT amplifier (Australia). Electrodes were placed according to the extended International 10–20 system, with reference electrodes placed at the subject’s nasal tip and ground electrode between Cz and CPz electrodes. The impedance of all electrodes was maintained below 5 kΩ, with a sampling rate of 500 Hz and acquisition time of no less than 8 min.

### 2.3 EEG pre-processing

The EEG data preprocessing was performed using EEGLAB. Continuous EEG was band-pass filtered at 0.1–30 Hz and notch filtered at 48–52 Hz, then segmented into 2-s epochs. Epoch-level artifact rejection combined automated thresholding and manual visual inspection. Epochs were automatically rejected if the peak-to-peak amplitude exceeded ±100 μV in any channel. In addition, all epochs were visually inspected, and any remaining epochs with residual artifacts (e.g., movement, muscle bursts, or transient electrode noise) were removed based on manual rating. Participants were excluded from further analysis if more than 30% of epochs were rejected, to ensure sufficient artifact-free data for stable source and connectivity estimation. ICA was applied to the epoched data. Artifact-related components were identified using ICLabel (15): an independent component was marked for removal if the ICLabel posterior probability was ≥0.90 for Eye or Muscle classes. Components flagged by ICLabel were subsequently reviewed using standard ICA characteristics (scalp topography, component time course, and power spectral density), and final component removal was confirmed by manual inspection, following published recommendations for ICA-based artifact correction (16).

### 2.4 EEG post-processing

Using the Brainstorm toolbox in MATLAB, Standardized Low-Resolution Brain Electromagnetic Tomography (sLORETA) was employed for source localization based on standardized current density from scalp EEG signals. A realistic head model constructed from the ICBM152 atlas was used, and the boundary element method (BEM) was applied to solve the forward problem, obtaining 6,340 voxel signals with a spatial resolution of 5 mm. Subsequently, nonlinear transformation was used to convert MNI coordinates to the Talairach coordinate system. The inverse operator was computed in a minimum-norm framework with free source orientations (three orthogonal components) and no depth weighting, and sLORETA was used as the standardization of the minimum-norm solution; regularization was applied via Tikhonov shrinkage with a fixed SNR = 3 (*λ*^2^ = 0.111), using an empirical noise covariance estimated from artifact-free resting EEG segments (17). Finally, 82 regions of interest (ROIs) were determined based on the Brodmann atlas. Lagged phase synchronization (LPS) between ROIs was calculated in the delta (0.5–4 Hz), theta (4–8 Hz), alpha (8–13 Hz), and beta (13–30 Hz) frequency bands to measure functional connectivity. LPS values were calculated separately for each epoch and then averaged across epochs for each participant. The frequency bands were defined *a priori* according to conventional EEG partitions and prior evidence in vascular cognitive impairment (VCI). A multidisciplinary expert-panel position paper on EEG/ERP markers in major VCI reported that the most consistent resting-state abnormalities are concentrated in delta, theta, and alpha rhythms, supporting analysis of delta (0.5–4 Hz), theta (4–8 Hz), and alpha (8–13 Hz) activity/connectivity in vascular populations (18). Theta-band activity has also been specifically linked to executive-control computations in frontal systems (19), while alpha-band oscillations are implicated in attentional gating/inhibitory control over information flow (20). In addition, beta-band synchronization (13–30 Hz) has been proposed to support maintenance of the current cognitive state (“status quo”), providing a complementary control-related band for connectivity analyses (21).

### 2.5 Feature selection

All participants with CAD were randomly split into a training set and a testing set at a ratio of 7:3. Feature selection was performed exclusively within the training set to avoid information leakage. First, univariate screening was conducted using independent-samples t tests to compare CAD patients with and without MCI. Variables showing significant between-group differences in the training set were retained as candidate features for subsequent selection. Second, the candidate features were further reduced using least absolute shrinkage and logistic regression with an L1 penalty. This step aimed to attenuate the impact of potential confounding and multicollinearity by shrinking less informative coefficients toward zero. Prior to model fitting, features were standardized using *Z*-score normalization; scaling parameters (mean and standard deviation) were estimated from the training data and applied within each cross-validation split. Model hyperparameters were tuned using five-fold stratified cross-validation with nested *Z*-score standardization, and the optimal regularization strength was selected by maximizing the cross-validated area under the receiver operating characteristic curve (AUC). The final set of features corresponded to predictors with non-zero coefficients under the optimal penalty and was carried forward for subsequent model development.

### 2.6 Interpretable machine learning

Eight machine learning methods were used to construct diagnostic models: k-nearest neighbors (KNN), decision tree, artificial neural network (ANN), support vector machine (SVM), gradient boosting, extra trees, random forest (RF), and extreme gradient boosting (XGBoost). In the training set, 5-fold cross-validation and grid search were used to adjust model parameters with the area under the curve (AUC) as the evaluation metric. After selecting the optimal parameters for each model, testing was performed on the test set. Model performance was evaluated using metrics such as AUC. To support clinical interpretability, we used SHAP primarily as a global explanation approach to quantify the relative contribution of each LPS-derived feature to model predictions in the training data. SHA*p* values were aggregated across subjects to rank connectivity features with the greatest overall impact on discrimination between MCI and non-MCI, and the directionality of effects (i.e., whether higher or lower connectivity increased the predicted probability of MCI) was summarized using SHAP dependence patterns. This global attribution was then mapped back to ROI-to-ROI pairs to facilitate neurophysiological interpretation of the model in terms of large-scale network alterations, while maintaining the intended role of SHAP as an explanatory tool rather than a standalone clinical decision rule.

### 2.7 Statistical analysis

Statistical analyses were performed using Python (Python Software Foundation, Wilmington, DE, United States). The Shapiro–Wilk test was employed to evaluate the normality of continuous variables. For continuous data with a normal distribution, results were expressed as mean ± standard deviation (*x* ± *s*) and compared across the three groups using one-way analysis of variance (ANOVA); *Post hoc* pairwise comparisons were conducted using Least Significant Difference (LSD) test for equal variances or Games–Howell test for unequal variances. For non-normally distributed continuous data, results were presented as median (lower quartile, upper quartile) [M(QL, QU)] and intergroup comparisons were performed using the Kruskal–Wallis *H* test, followed by Dunn’s test for pairwise comparisons. Categorical variables (e.g., gender, presence of diabetes) were summarized as counts (percentages) and compared among the three groups using the Pearson’s chi-square (*χ*^2^) test. The statistical significance level was set at *p* = 0.05, false discovery rate (FDR) correction was applied in the EEG statistical analyses to control for multiple testing.

## 3 Results

A total of 53 patients with primary hypertension without CAD and 117 patients with CAD comorbid with primary hypertension were included (49 with MCI and 68 without MCI). There were no significant between-group differences among the three groups in age, sex, years of education, hypertension grade, or the presence of diabetes or hyperlipidemia (all *p* > 0.05; Table 1). Because coronary stenosis was not assessed in some non-CAD hypertensive patients who were asymptomatic and without major risk factors, stenosis data were reported only for the two CAD groups, in which stenosis severity did not differ significantly between the MCI and non-MCI subgroups (*p* > 0.05; Table 1). In addition, the MCI group exhibited lower MoCA scores compared with the other two groups (*p* < 0.05; Table 1).

**Table 1:** General data of the each groups.

Characteristic	Non-CAD hypertension (*n* = 53)	CAD without MCI (*n* = 68)	CAD with MCI (*n* = 49)	*P*-value
Age (years), mean ± SD	68.08 ± 5.08	66.28 ± 4.23	67.51 ± 4.82	0.17
Male sex, *n* (%)	20 (37.7)	22 (32.4)	18 (36.7)	0.80
Disease duration of CAD (months), mean ± SD	–	53.46 ± 29.39	61.24 ± 35.14	0.194
Education ≤12 years, *n* (%)	29 (54.7)	49 (72.1)	32 (65.3)	0.14
Hypertension grade, *n* (%)				0.89
Grade 1	27 (50.9)	30 (44.1)	22 (44.9)	
Grade 2	18 (34.0)	27 (39.7)	17 (34.7)	
Grade 3	8 (15.1)	11 (16.2)	10 (20.4)	
Diabetes, *n* (%)	16 (30.2)	18 (26.5)	16 (32.7)	0.76
Hyperlipidemia, *n* (%)	17 (32.1)	22 (32.4)	18 (36.7)	0.85
Coronary stenosis degree, *n* (%)				0.732
Moderate stenosis	–	41 (60.3)	28 (57.1)	
Severe stenosis	-	27 (39.7)	21 (42.9)	
Retained artifact-free EEG epochs, mean ± SD	159.81 ± 22.85	159.75 ± 21.4	153.92 ± 19.08	0.268
MoCA score, mean ± SD	28.02 ± 1.39	27.94 ± 1.40	22.53 ± 1.72	<0.05

### 3.1 Changes of LPS in CAD patients

**Figure 1:**
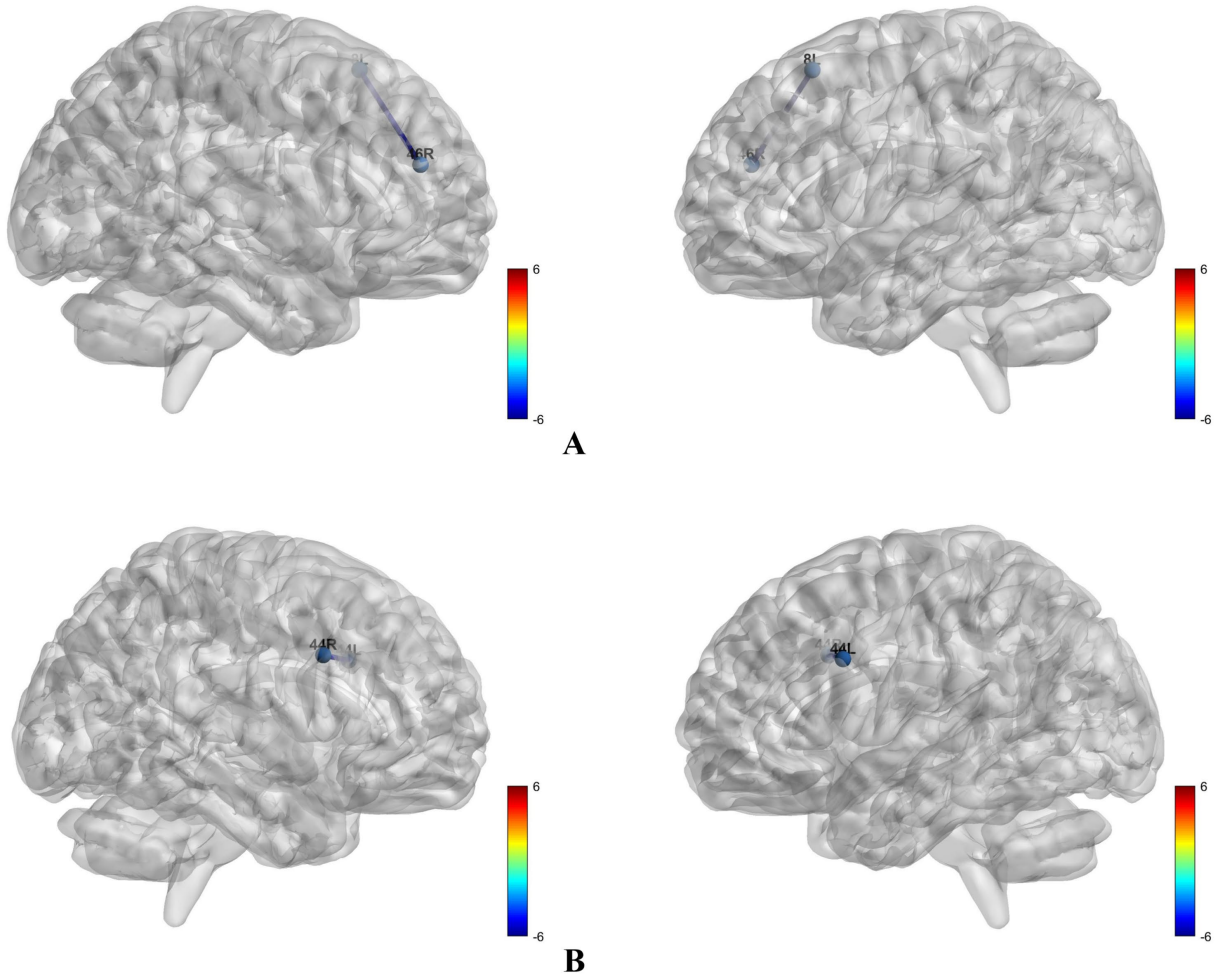
Brain regions with altered lagged phase synchronization in different frequency bands: **(A)** Brain regions with altered lagged phase synchronization in the alpha band; **(B)** brain regions with altered lagged phase synchronization in the beta band.

Compared with primary hypertension, cognitively normal CAD patients showed decreased LPS in 8L-46R (alpha band) (*p* < 0.05, Figure 1A) and 44L-44R (beta band) (*p* < 0.05, Figure 1B).

### 3.2 Changes of LPS in MCI patients

**Figure 2:**
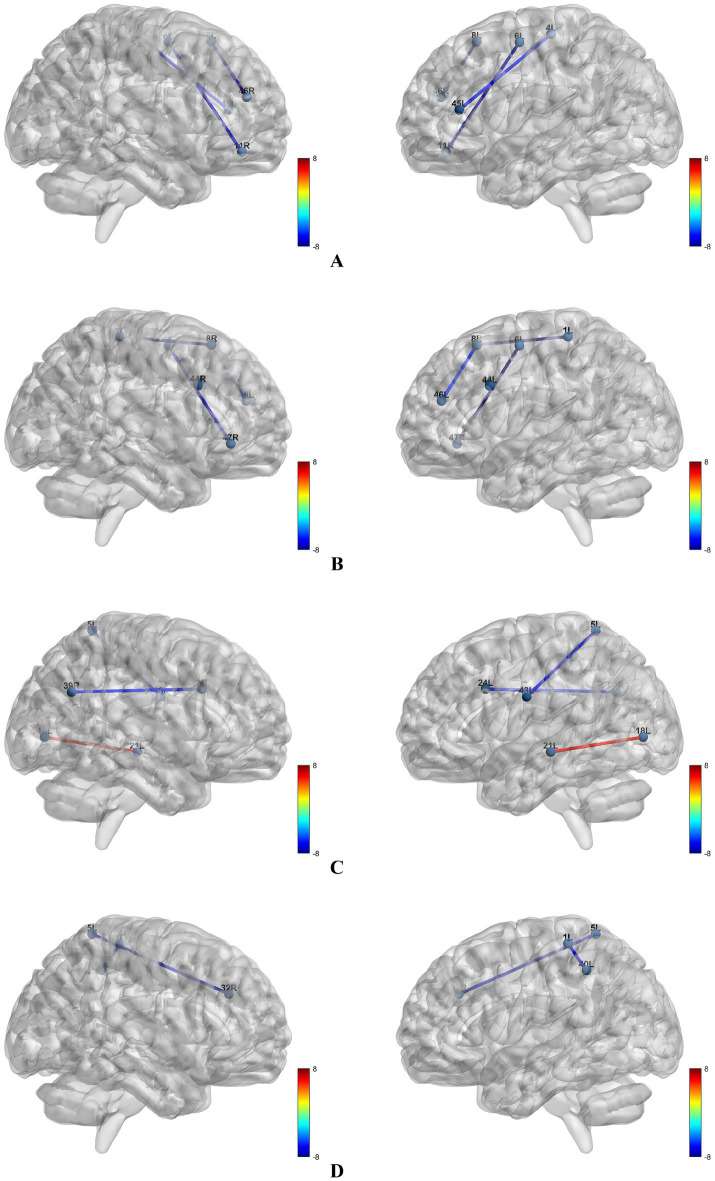
Brain regions with altered lagged phase synchronization in different frequency bands. **(A)** Brain regions with altered lagged phase synchronization in the *α* band; **(B)** brain regions with altered lagged phase synchronization in the β band; **(C)** brain regions with altered lagged phase synchronization in the *δ* band; **(D)** brain regions with altered lagged phase synchronization in the *θ* band.

Compared with cognitively normal CAD patients, CAD patients with MCI exhibited decreased LPS in 8L-46R, 6L-11R, and 4L-45L (alpha band) (*p* < 0.05, Figure 2A); 1L-8R, 8L-46L, 44L-44R, and 6L-47R (beta band) (*p* < 0.05, Figure 2B); 1L-40L and 5L-32R (theta band) (*p* < 0.05, Figure 2D). In the delta band, the MCI group showed increased LPS in 18L-21L but decreased LPS in 5L-43L and 24L-39R compared to cognitively normal CAD patients (*p* < 0.05, Figure 2C).

### 3.3 Feature selection

**Figure 3:**
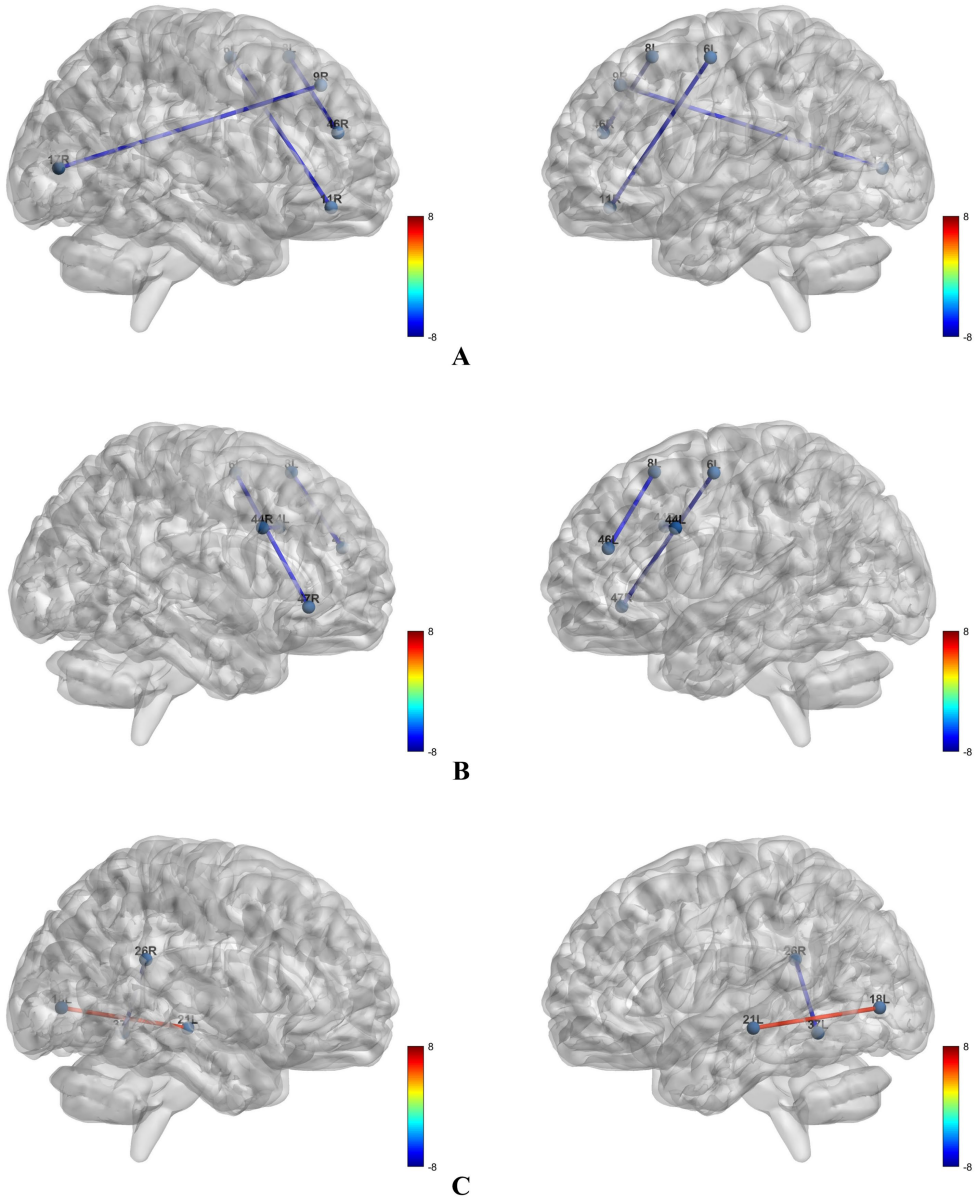
Brain regions with altered lagged phase synchronization in different frequency bands. **(A)** Brain regions with altered lagged phase synchronization in the α band; **(B)** brain regions with altered lagged phase synchronization in the β band; **(C)** brain regions with altered lagged phase synchronization in the δ band.

**Figure 4:**
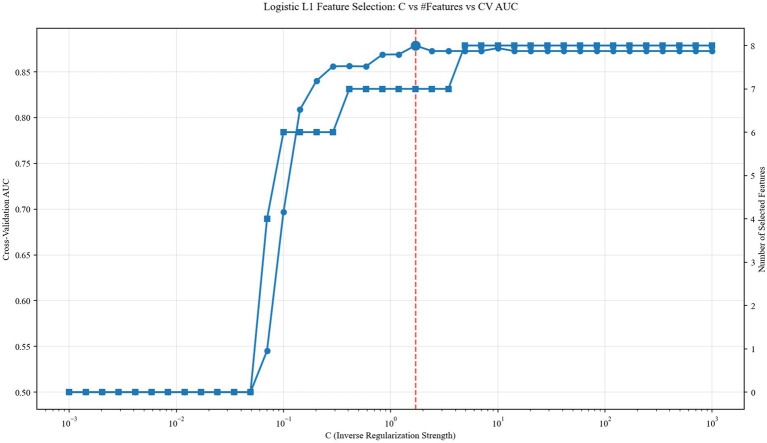
L1-penalized logistic regression feature selection results.

The CAD cohort was randomly split into a training set and a testing set at a 7:3 ratio. In the training set, univariate screening using independent-samples t tests identified multiple LPS features that differed between CAD patients with MCI and cognitively normal CAD patients. Specifically, compared with cognitively normal CAD patients, the MCI group showed reduced LPS in 8L–46R, 6L–11R, and 9R–17R within the alpha band (all *p* < 0.05; Figure 3A), and reduced LPS in 8L–46L, 44L–44R, and 6L–47R within the beta band (all *p* < 0.05; Figure 3B). In the delta band, CAD patients with MCI exhibited increased LPS in 18L–21L and decreased LPS in 26R–37L relative to cognitively normal CAD patients (all *p* < 0.05; Figure 3C). No significant between-group differences were observed in the theta band (*p* > 0.05). Overall, the training-set findings were largely consistent with those obtained in the full cohort; however, several associations evident in the overall sample were not detected in the training subset, likely reflecting reduced statistical power after sample splitting. Candidate features were subsequently subjected to logistic regression with five-fold cross-validation for hyperparameter tuning. The optimal regularization parameter was identified at *C* = 1.194, yielding the highest cross-validated AUC (0.863, Figure 4). Under this optimal penalty, seven features with non-zero coefficients were retained: 18L–21L (*δ*), 8L–46L (*β*), 8L–46R (*α*), 6L–11R (α), 26R–37L (δ), 9R–17R (α), and 44L–44R (β). These selected features were carried forward for subsequent model construction.

### 3.4 Interpretable machine learning

**Figure 5:**
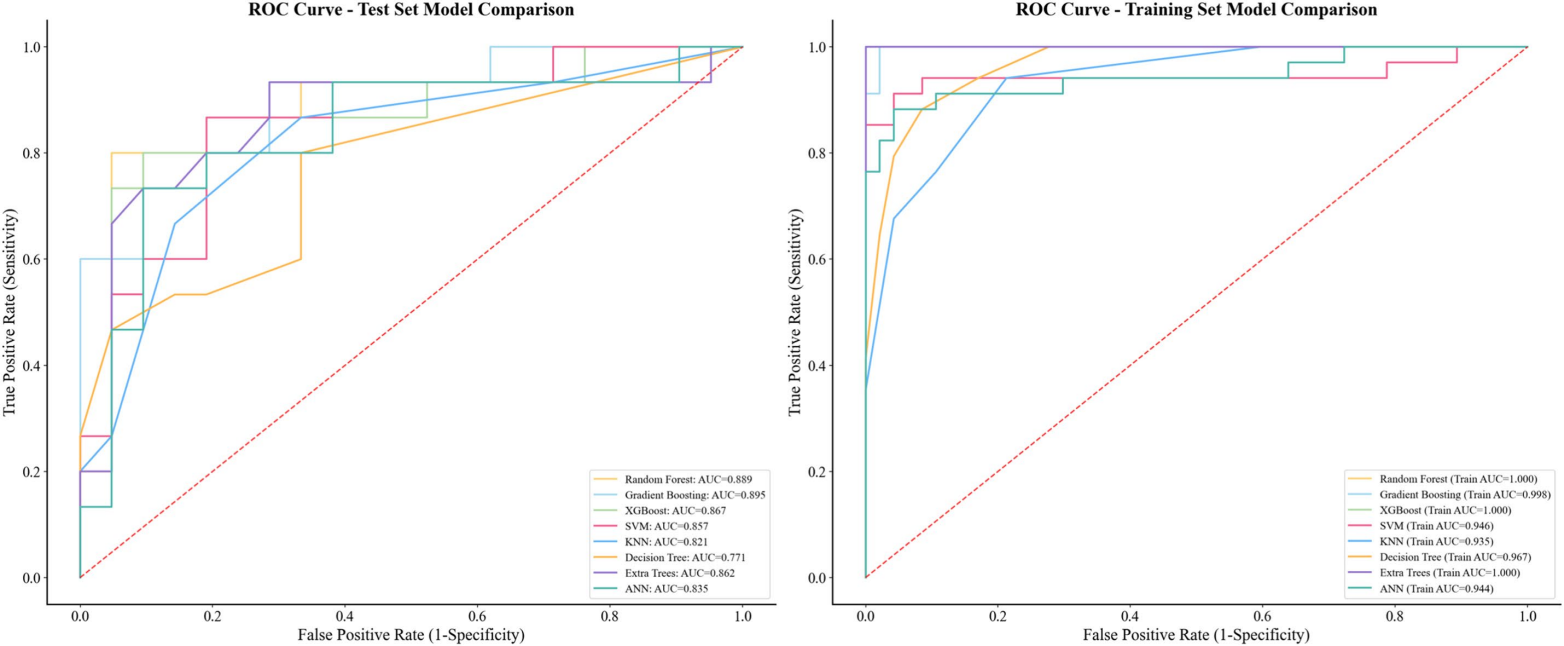
Area under the curve (AUC) of the 8 models in the test set and training set.

**Figure 6:**
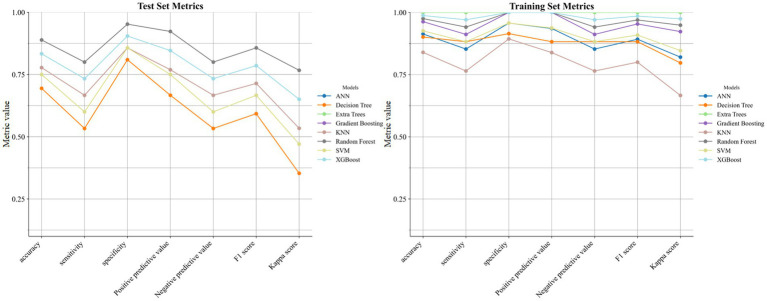
Supplementary evaluation indicators of the 8 models.

**Figure 7:**
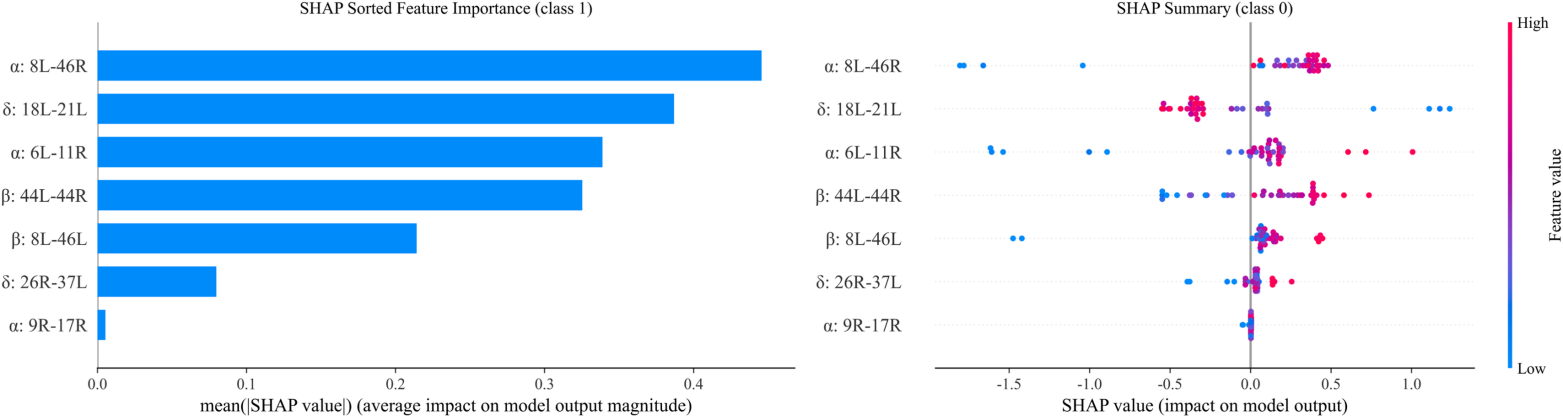
Contribution of different EEG impairments to the diagnosis of cognitive impairment.

Machine learning results showed that among the 8 models in the test set, the Gradient Boosting model had the best performance in cognitive impairment diagnosis with an AUC of 0.895 (95% CI:0.809–0.981, Figure 5), and the evaluation metrics of other models are shown in Figure 6. SHAP value analysis revealed that the top 5 EEG LPS indicators most meaningful for cognitive impairment diagnosis were: decreased alpha band 8L-46R, increased delta band 18L-21L, decreased alpha band 6L-11R, decreased beta band 44L-44R, and decreased beta band 8L-46L (Figure 7).

## 4 Discussion

This investigation sought to delineate the electrophysiological underpinnings of MCI in elderly patients with CAD through source-localized EEG and machine learning approaches. The core findings indicate that CAD induces specific reductions in LPS within alpha and beta frequency bands, predominantly involving frontal regions, with MCI exacerbating these disruptions across multiple bands and networks. The high-performing Gradient Boosting model identifies that the key connectivity alterations driving cognitive decline lie in decreased activity in the alpha band (8L-46R).

The observed decrease in alpha-band LPS between BA 8 L and 46R in cognitively normal CAD patients compared to non-CAD hypertensive controls suggests impaired functional integration between premotor and dorsolateral prefrontal cortices, which are critical for executive control and working memory (22). This alteration likely stems from CAD-associated cerebral hypoperfusion, leading to reduced neural efficiency in these regions, consistent with vascular mechanisms that compromise metabolic support for synchronized oscillatory activity. Similarly, the beta-band reduction in BA44L-44R connectivity implies disrupted bilateral opercular networks involved in language and motor planning, potentially reflecting microvascular damage that affects high-frequency oscillations essential for rapid information processing (23, 24). In CAD patients with MCI, the expanded alpha-band decrements, including BA6L-11R and BA4L–45L, extend to premotor-orbitofrontal and primary motor-pars triangularis pathways, indicating broader frontal lobe involvement that may underlie deficits in decision-making and motor-cognitive integration (25). These patterns align with theoretical models positing that chronic ischemia in CAD progressively erodes prefrontal integrity, thereby amplifying vulnerability to cognitive impairment (2).

Furthermore, the beta-band changes in MCI, such as diminished LPS in BA1L-8R and BA8L–46L, highlight somatosensory-premotor and premotor-prefrontal disconnections, which could contribute to attentional lapses and executive dysfunction through impaired sensory-motor feedback loops (26). The theta-band reductions in BA1L–40L and BA5L-32R point to disrupted somatosensory-parietal and association-cingulate networks, potentially linked to memory retrieval impairments, as theta rhythms facilitate hippocampal-prefrontal interactions often compromised in vascular pathologies (12). Notably, the delta-band findings in MCI—elevated LPS in BA18L–21L alongside decreases in BA5L–43L and BA24L-39R—suggest compensatory hyper-synchronization in visual-temporal areas contrasted with hypo-connectivity in somatosensory-gustatory and cingulate-angular pathways, reflecting adaptive yet inefficient low-frequency reorganization amid progressive neurodegeneration (27). SHAP analysis underscores the diagnostic salience of these features, with the alpha-band BA8L-46R decrement emerging as the paramount contributor, implying that frontal dysconnectivity serves as a primary electrophysiological hallmark of MCI progression in CAD.

These interpretations are bolstered by the machine learning framework, where the Gradient Boosting model’s superior AUC of 0.895 in distinguishing MCI from non-MCI CAD patients highlights the predictive power of multi-band LPS metrics. The model’s emphasis on decreased alpha and beta frontal connectivities, alongside delta alterations, suggests a hierarchical impact wherein high-frequency disruptions initiate cognitive vulnerability, subsequently amplified by low-frequency compensatory shifts (28). This mechanism may involve endothelial dysfunction in CAD, promoting neuroinflammation and amyloid deposition that further desynchronizes neural ensembles. Collectively, these analyses support our hypothesis that CAD is associated with targeted electrophysiological alterations, and that MCI status within CAD is associated with more extensive network fragmentation; mechanistic inferences regarding vascular-mediated stress require direct physiological validation.

In comparison to extant literature, our identification of alpha and beta LPS reductions in frontal regions among CAD patients resonates with prior EEG studies documenting diminished high-frequency connectivity in vascular cognitive impairment, where hypoperfusion correlates with executive deficits. For instance, investigations into post-coronary syndrome cognitive changes have reported similar beta-band attenuations in opercular areas, attributing them to ischemic microvascular injury (7). However, discrepancies arise in delta-band dynamics; while some reports indicate uniform delta increases in MCI as markers of cortical slowing (12), our mixed findings—elevation in temporal-visual links versus reductions elsewhere—suggest CAD-specific regional heterogeneity, possibly due to comorbid hypertension modulating low-frequency responses differently than in isolated MCI cohorts. This contrasts with functional MRI-based research showing generalized default mode network hypo-connectivity in CAD-related dementia (9), yet our EEG approach offers superior temporal resolution, revealing band-specific nuances absent in hemodynamic measures. Novel contributions of this work include the application of sLORETA for precise source localization in CAD-MCI, filling gaps in prior scalp-level EEG analyses that overlooked subcortical contributions (29), and the integration of interpretable machine learning via SHAP to quantify feature importance, extending beyond descriptive comparisons in similar vascular studies. Unlike machine learning models predicting Alzheimer’s progression from EEG (10), our focus on CAD-specific MCI addresses an understudied intersection, resolving inconsistencies in literature by controlling for hypertension and employing rigorous multiple comparison corrections. These advancements bridge methodological limitations in earlier works, such as smaller samples or lack of source reconstruction (11, 22), thereby enhancing generalizability to elderly cardiovascular populations.

The academic significance of these findings lies in advancing theoretical models of vascular-neural interplay, demonstrating how CAD precipitates MCI through targeted connectivity disruptions that can be quantified non-invasively via EEG. Clinically, this paves the way for EEG-based biomarkers to facilitate early MCI screening in CAD patients, potentially informing tailored interventions like cognitive training or vascular risk management to mitigate progression. From a technological standpoint, the validated Gradient Boosting model offers a scalable tool for precision diagnostics, with implications for integrating EEG analytics into routine cardiac care. Future research could explore therapeutic applications, such as neuromodulation targeting identified frontal networks, to restore connectivity and preserve cognition.

Notwithstanding these strengths, the study’s retrospective design introduces potential selection bias, as reliance on existing clinical data may overlook unrecorded confounders like lifestyle factors. The moderate sample size (*n* = 170 total), while sufficient for intergroup comparisons, limits statistical power for subgroup analyses, potentially underestimating rarer connectivity patterns. Furthermore, while the MoCA is a validated instrument for MCI screening, our reliance on a single assessment tool may not fully capture the complexity of multidomain cognitive deficits. Methodological constraints, including the focus on resting-state EEG without task-specific paradigms, may not capture dynamic cognitive processes, and the single-center recruitment restricts generalizability to diverse ethnic or socioeconomic groups. Although an independent test set was used, the relatively limited sample size and the high dimensionality of EEG connectivity features may still introduce a risk of overfitting. Multicenter studies with larger cohorts and multimodal imaging could help address these limitations and enhance external validity.

In summary, this study characterizes EEG connectivity patterns associated with MCI status in an elderly CAD cohort, and machine learning highlights candidate features for classification that may inform vascular neuroscience. Prospective longitudinal investigations incorporating interventions could further validate these biomarkers and guide preventive strategies in at-risk elderly populations.

## 5 Conclusion

This study demonstrates that CAD in elderly patients is associated with specific electrophysiological alterations, characterized by reduced lagged phase synchronization in the alpha and beta bands within frontal networks. These alterations are more pronounced in CAD patients with MCI and extend to the theta and delta bands, consistent with a progression toward broader neural network fragmentation. By integrating source-localized EEG with interpretable machine learning, we identify key connectivity alterations—particularly in premotor–prefrontal pathways—as candidate biomarkers for MCI classification within CAD, supporting a vascular-relevant interpretation that requires multimodal confirmation. These findings underscore the potential for non-invasive EEG-based tools to enable early detection and intervention and motivate longitudinal studies to test whether interventions targeting vascular risk or cognitive function are associated with favorable changes in these EEG connectivity features.

## Data Availability

The original contributions presented in the study are included in the article/supplementary material, further inquiries can be directed to the corresponding author.
